# Defining the population of adolescents in need of comprehensive transitional care based on diagnosis, visit frequency, and disease complexity

**DOI:** 10.1371/journal.pone.0339721

**Published:** 2026-01-27

**Authors:** Maj Beldring Henningsen, Kirsten Arntz Boisen, Pi Vejsig Madsen, Andreas Jensen, Helene Kildegaard, Charlotte Blix, Jakob Lorentzen, Line Hartvig Cleemann, Trine Spiegelhauer, Christine Dahl, Anne Elisabeth Bjerrum, Ann-Sophie Buchardt, Lone Graff Stensballe

**Affiliations:** 1 Mary Elizabeth’s Hospital, Copenhagen University Hospital Rigshospitalet, Copenhagen, Denmark; 2 Department of Pediatrics and Adolescent Medicine, Copenhagen University Hospital Rigshospitalet, Copenhagen, Denmark; 3 Department for Neuroscience, University of Copenhagen, Copenhagen, Denmark; 4 Department of Growth and Reproduction, Copenhagen University Hospital Rigshospitalet, Copenhagen, Denmark; 5 Department of Pediatric Surgery, Copenhagen University Hospital Rigshospitalet, Copenhagen, Denmark; 6 Institute of Clinical Medicine, University of Copenhagen, Copenhagen, Denmark; National Research Centre, EGYPT

## Abstract

Healthcare transition from pediatrics to adult care is a critical yet challenging process for adolescents with long-term medical conditions. This population-based cohort study aims to present a replicable method to identify and quantify adolescents in need of comprehensive transitional care. Using data from Danish national health registers, disease complexity was categorized by expert clinicians based on diagnoses indicative of a need for comprehensive transitional care and transfer to specialized adult healthcare. The study identified 4,677 adolescents requiring comprehensive transitional care from a background population of 418,994 Danish adolescents aged 16–17 years, corresponding to 1.1%. Analysis of outpatient visit data from tertiary hospitals revealed variability in the proportion of adolescents with comprehensive transitional care needs across Denmark’s four tertiary hospitals. For instance, 11.6% of outpatient visits at Aalborg University Hospital involved a comprehensive transition-requiring diagnosis, compared to 26.7% at Copenhagen University Hospital. While the method is intentionally specific and focused on adolescents with the most complex conditions, it offers a scalable framework that could be applied across broader clinical settings. We illustrate this by also applying the method within a pediatric department-based setting. This study provides al replicable framework to assess transition care needs at a population level, primarily identifying adolescents with the most complex conditions. Broader implementation across clinical settings may refine and inform equitable transitional strategies.

## Introduction

### Background and rationale

The transfer from pediatric to adult healthcare is a critical phase for adolescents with chronic medical conditions [1]. This transition involves shifting from family-centered pediatric care to adult-oriented systems, which introduces changes in providers, clinical environments, expectations, and legal frameworks, as well as increased demands for self-management [1,2].

A successful transition is a planned process in time and context that ensures continuity of care, promotes the development of self-management skills (including logistical planning) and supports the overall well-being of the adolescent throughout the process [1,3–5]. National and international guidelines highlight the need for early initiation and developmentally appropriate care to facilitate this process [6–8].

For the European region, WHO has recently reported a need for a data-driven approach to ensure sufficient health service based on specific personal needs, rather than the one-size-fits-all approach [9]. Similarly, using routine healthcare data to estimate the age at which adolescents transfer from pediatric to adult care helps improve planning by moving beyond arbitrary age cut-offs [10]. A prerequisite for such planning is identifying adolescents with the highest anticipated healthcare needs

In Denmark, transitional care has been recognized as a core responsibility in pediatrics since 2017, as outlined by the Danish Health Authority [11]. The authority has also issued recommendations for transitioning from pediatric to adult care in hospital settings [7]. Additionally, transitional care is a key component of the Danish guidelines for an adolescent-friendly healthcare system [4].

Knowing the prevalence of long-term medical conditions among adolescents is essential for organizing, scaling, and evaluating transitional care at national and regional hospital levels. Internationally, reported rates of long-term medical conditions among children and adolescents vary widely [12–14], and a systematic review reported prevalence ranging from 0.22% to 44% [15]. This broad range underscores the absence of standardized, diagnosis-based methods, like what is presented in the present study, to determine which adolescents require transfer to specialized adult care. In Denmark, estimates also differ depending on definitions and data sources, with up to 20% of adolescents potentially having a chronic somatic or mental health condition [16,17], 15% report long-term conditions affecting daily life [18], and severe chronic somatic illness has been estimated at 4.7% based on ICD-10 codes [19]. A consistent approach for identifying those specifically requiring specialized adult healthcare remains lacking.

The importance of a successful transition to adult healthcare for adolescents is widely recognized [20,21]. Although international guidelines suggest that all adolescents could benefit from transitional support [3,8], limited resources necessitate prioritizing adolescents with the greatest need for coordinated and comprehensive care [22]. To achieve this, it is essential to first evaluate which diagnoses may require transfer to specialized adult healthcare. A quantitative, diagnosis-based approach can help determine which conditions are most likely to require ongoing specialized management into adulthood.

While previous studies have assessed transition needs within individual subspecialties, such as pediatric surgery, urology, and congenital heart disease [23–25], there is limited evidence on the broader transition population across the full spectrum of pediatric specialties. Addressing this gap is essential for developing tailored interventions and informing service planning.

In Denmark, comprehensive population and health registers provide a unique infrastructure for national cohort studies, enabling accurate linkage of individual-level data [26,27]. Despite recognition of the need for transitional care, no standardized method exists for identifying adolescents who require specialized adult health care. This study therefore aims to develop and test a replicable, diagnosis-based framework to identify this population at the national level by combining expert-validated diagnostic criteria with outpatient visit data.

## Methods

### Study design and setting

The study was a population-based cohort study leveraging comprehensive data from Danish nationwide health registers.

### Data sources

The background population included all adolescents 12- to 17-year-old living in Denmark in the period 2019–2022. Information on sex and date of birth was extracted from the Central Personal Register and the Medical Birth Register [28]. Information on residence and vital status was extracted from the Central Person Register [27]. Finally, elective outpatient contacts at tertiary hospitals were identified using the Danish National Patient Register (DNPR) [26].

### Identification of adolescents in need of comprehensive transitional care

The procedure for identifying adolescents in need of comprehensive transitional care was outlined as follows.

First, the age period 12–17 was chosen, since these are the defining years in terms of transitioning from the pediatric to adult healthcare system, with most adolescents transferring from pediatric care at the age of 18. The study period 2019–2022 was chosen to exploit the most recent data available. Adolescents were included if resident in Denmark at any point during the study period and were excluded upon death or emigration.

Because individuals aged 12–15 during the study period could age into the 16–17 group, the background population included all adolescents who reached age 16 or 17 between 2019 and 2022, regardless of their age at study entry.

In Denmark, healthcare is delivered through primary, secondary, and tertiary sectors. The primary analysis focused on outpatient visits at the four tertiary hospitals providing highly specialized care (Rigshospitalet, Aarhus University Hospital, Odense University Hospital, and Aalborg University Hospital). The definition of elective outpatient visits is provided in S1 Table. This is referred to as the tertiary hospital approach. Our method also included a diagnosis criterion. The diagnoses associated with adolescents potentially requiring transfer to specialized adult care were identified based on prior work, which used ICD-10 diagnosis codes indicating severe chronic disease [29].

### Clinical expert evaluation

The preliminary list of diagnoses was then thoroughly evaluated and refined by specialized expert clinicians, including clinical pediatric experts across subspecialties and surgeons from various fields, such as pediatric surgery. In the refinement process, we decided to exclude certain specialties, such as orthopedic surgery, otorhinolaryngology, ophthalmology, and dermatology, as these specialties cover both pediatric and adult patients in Denmark meaning no transfer needed.

### Categorizing diagnoses related to need of comprehensive transitional care

Inspired by a previous study, the clinical experts also categorized diagnoses into four levels (A-D) to reflect the varying degrees of complexity [30].

A Category A included adolescents seen 1–2 times annually, with simpler conditions like asthma or uncomplicated congenital heart disease.
B Category B involved cases requiring a single medical specialist, such as type 1 diabetes or cystic fibrosis.
C Category C covered more complex conditions involving multiple specialties, like organ transplants or rare complex diseases.
D Category D was reserved for the most complex conditions requiring extensive multidisciplinary care and several specialties, such as neuromuscular diseases.

Notably, a single diagnosis could fall into multiple categories depending on complexity. For example, cerebral palsy may be classified under different levels depending on the complexity.

To further refine this classification, we applied a “lower bound” and “upper bound” scenario:

- The “lower bound” scenario assumes the least complex interpretation of each diagnosis, classifying conditions at the mildest plausible level.
- The “upper bound” scenario assumes the highest possible complexity for each diagnosis, capturing cases that require the most comprehensive care.

The finalized list of ICD-10 codes related to comprehensive transitional care needs, along with their assigned complexity categories (A–D), is provided in the supporting material (S2 Table). Additionally, a separate category (S) was introduced for pediatric surgical conditions requiring transition, as determined through expert consultation.

### Alternative approach: Paediatric department-based identification

Acknowledging that transitional care also exists outside of tertiary hospitals, an alternative pediatric department approach was used in addition to the tertiary hospital approach described above. This secondary approach included outpatient visits at Danish pediatric departments for the same background population of adolescents aged 12–17 years. The full list of Danish pediatric departments is provided in the supporting material (S3 Table).

### Stepwise cohort grouping

Four nested groups of adolescents were defined with increasing specificity, ultimately identifying the final study population. Throughout the manuscript these cohort groups are denoted sub-cohorts 1–4:

**Sub-cohort 1.** Adolescents aged 12–17 with at least one outpatient visit at a tertiary Danish hospital during 2019–2022.

**Sub-cohort 2.** Adolescents aged 12–17 with at least one outpatient visit at a tertiary Danish hospital *related to a condition expected to require comprehensive transitional care* during 2019–2022, according to the list of diagnoses (S2 Table).

**Sub-cohort 3.** Adolescents aged 12–17 with at least *two* outpatient visits at a tertiary Danish hospital *related to a condition expected to require comprehensive transitional care* during 2019–2022, according to the list of diagnoses (S2 Table). A minimum of two outpatient visits was chosen to ensure that identified adolescents had ongoing care needs rather than single diagnostic assessments.

**Sub-cohort 4.** Adolescents aged 12–17 with at least two outpatient visits *related to a condition expected to require comprehensive transitional care* during 2019–2022, according to the list of diagnoses (S2 Table), *with at least one visit occurring between the ages of 16 and 17 years.*

This stepwise approach was designed to systematically refine the study population by progressively applying more specific criteria. Each sub-cohort reflects a group more likely to require comprehensive transitional care, facilitating comparisons, and ensuring that the final population represents those most in need of comprehensive transition.

### Identification of adolescents primarily followed by pediatric surgeons in need of comprehensive transitional care among adolescents

Additionally, a pediatric surgical transition population was defined. This population was established using a similar four-step approach as described in the stepwise cohort grouping, but it was limited to adolescents aged 12–15 years, as surgical patients in Denmark typically transfer to adult care at age 16. The populations are referred to as surgical cohorts 1–4.

The identification of surgical diagnoses associated with an expected need for transitional care was guided by pediatric surgeons. The categorization (denoted S in S2 Table) ensured that only diagnoses linked to an expected need for comprehensive transitional care were included, as described for the tertiary hospital population.

For the final pediatric surgical study cohort, individuals were required to have had at least two outpatient visits related to a surgical diagnosis associated with an expected need for transitional care at a tertiary Danish hospital between 2019 and 2022. Of these visits, we required at least one to have occurred when the adolescent was between the ages of 14 and 15 years.

### Data access and confidentiality

Data for this study were accessed for research purposes from 20 February 2023 to 20 October 2024. Data were pseudonymized, and groups with fewer than five individuals were not disclosed to ensure confidentiality. The data supporting this study’s findings are available from Statistics Denmark but are not publicly accessible due to licensing restrictions. For assistance with data access under Statistics Denmark’s permissions, contact the corresponding author.

Data processing and descriptive statistics were carried out in the statistical software program R, using the tidyverse package [31,32].

## Results

### Stepwise algorithm

A stepwise filtering process was used to narrow down the population, as outlined in the methods section. This approach ensured the identification of adolescents with a comprehensive need for transitional care based on diagnoses and outpatient visits.

**Fig 1:**
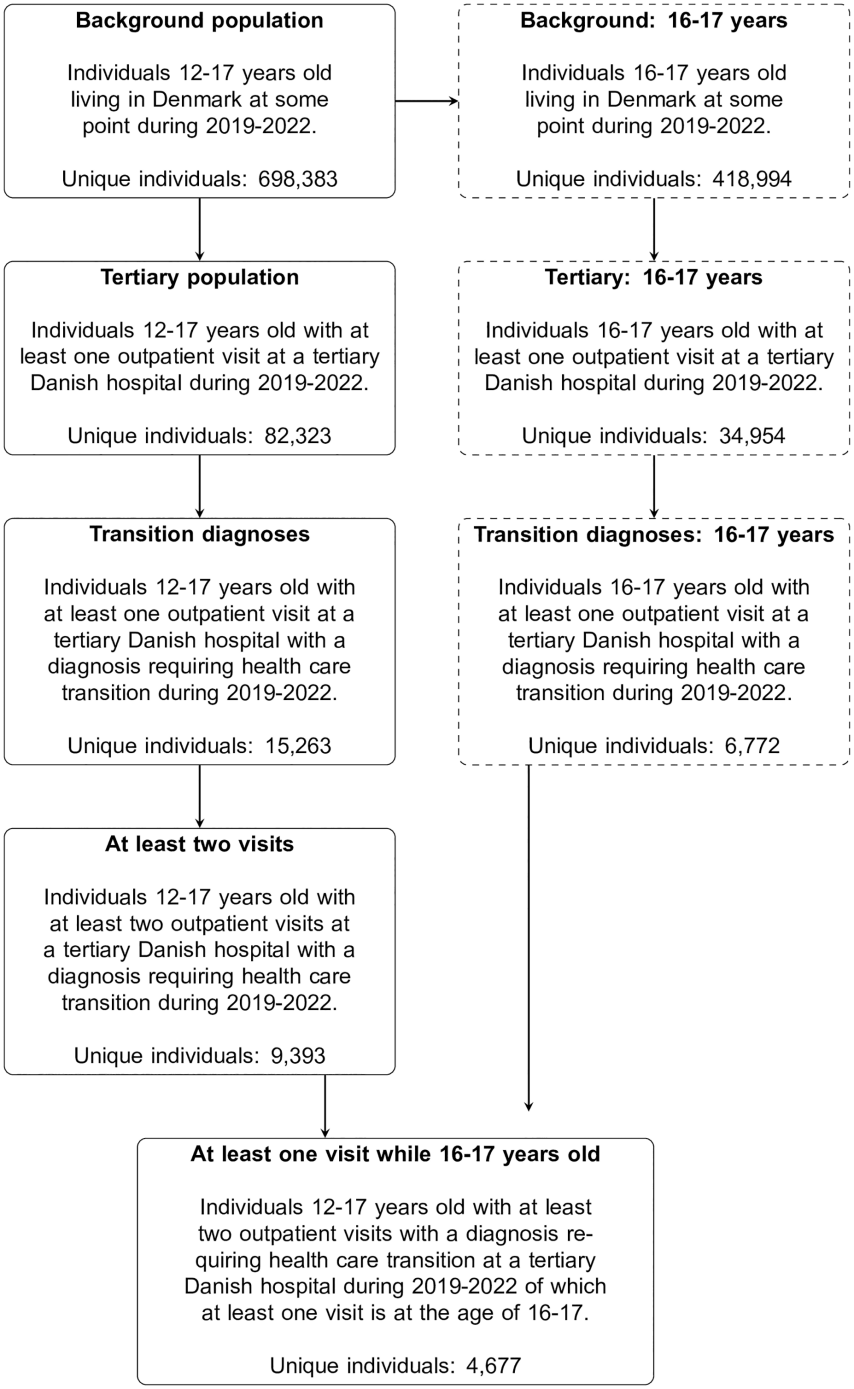
Flowchart illustrating the number of unique adolescents in each of the sub-cohort steps (tertiary hospital-based). The left-hand side describes the distribution of adolescents in each step. The right-hand side of the figure is included to acknowledge the fact that not all adolescents in the cohort reached age 16-17 years during the study period.

The stepwise filtering process is illustrated in Fig 1, which includes the subpopulation of adolescents aged 16–17 years to acknowledge that not all in the full study cohort reached this age during the study period. The overall person-years spent in Denmark by 12- to 17-year-olds during 2019–2022 amounted to 1,645,765 person-years, with 548,977 person-years spent by 16- to 17-year-olds. The observed variation in person-years across age groups suggests differences in healthcare utilization patterns, which could influence the transitional care needs of adolescents.

In the background population of 698,383 unique 12- to 17-year-olds, 82,323 adolescents (11.8%) had at least one elective outpatient visit at a tertiary hospital in Denmark during 2019–2022 (Fig 1). These adolescents accounted for a total of 272,973 outpatient visits. Among these outpatient visits, 66,638 (24.4%) included at least one diagnosis requiring transfer to specialized adult healthcare.

Of the 1,981 unique 12- to 15-year-olds who were seen at a tertiary hospital with surgical-related diagnoses with an expected need of comprehensive transition, 616 had at least two visits, including one while aged 14–15 years.

Among the adolescents with at least one outpatient visit at a tertiary hospital, 15,263 individuals (2.2% of the population) were diagnosed at least once with one of the diagnoses associated with an expected need of comprehensive transitional care (diagnoses listed in S2 Table), with 9,393 (1.4%) individuals having at least two registrations of such diagnoses (Fig 1). Applying the diagnosis-based criteria excluded 177,583 outpatient visits at tertiary hospitals. The primary diagnoses associated with these excluded visits were identified (S4 Table) with diagnosis Z01 – an encounter for general examination without complaint, being the most common exclusion diagnosis, accounting for 30% of the excluded visits.

### Analyses restricted to 16- to 17-year-old adolescents

Restricting the population to adolescents living in Denmark between the ages of 16 and 17 during the period 2019–2022, we found that, of the background population of 418,994 unique 16- to 17-year-olds, 34,954 had at least one outpatient visit at a tertiary hospital (Fig 1). Among these, a subset of 6,772 adolescents (1.6%) was diagnosed with at least one condition associated with an expected need for comprehensive transitional care. This proportion was comparable to that observed in the broader 12- to 17-year-old population, suggesting consistent needs across age groups.

**Fig 2:**
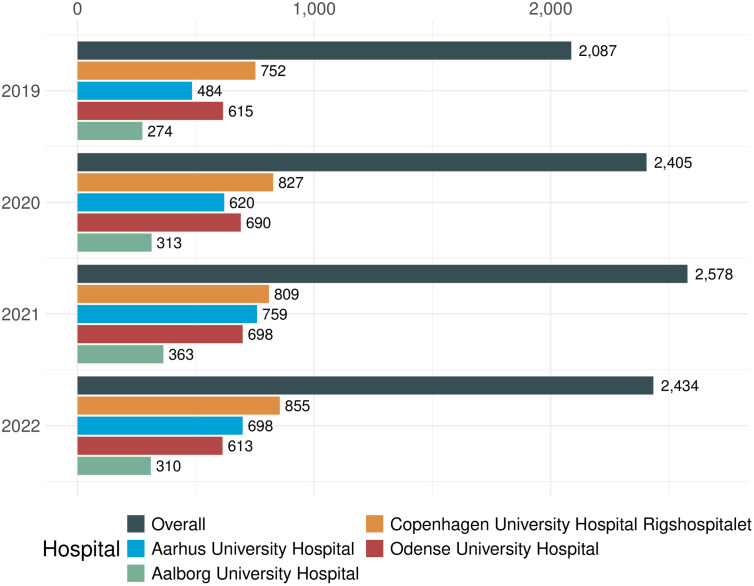
Distribution of adolescents, 16- to 17-year-old, with outpatient visits relevant to comprehensive transitional care across tertiary hospitals. No decrease in the number of adolescents was observed during the COVID-19 pandemic from 2020 to 2021. Note that adolescents may be associated with multiple hospitals, so the totals do not sum across hospitals. Diagnoses with surgical transition needs are indicated separately in the supporting materials.

The total of 16- to 17-year-old individuals with at least one outpatient visit for a diagnosis associated with an expected need of transitional care at a tertiary hospital remained stable over the period 2019–2022, ranging between 2,087 and 2,578 unique adolescents per calendar year. This consistency suggests that the need for transitional care remains constant, even during disruptions such as the COVID-19 pandemic. The overall and hospital-specific development is illustrated in Fig 2.

The final population, based on outpatient visits at tertiary hospital after applying all the criteria listed in the methods section, comprised 4,677 adolescents (1.1%) with at least two outpatient visits for conditions associated with an expected need for transitional care, with at least one visit occurring while they were aged 16–17 years.

### Disease complexity

Table 1 illustrates the distribution of adolescents aged 16–17 across Danish tertiary hospitals, categorized by degree of complexity, ranging from A (minimal complexity) to D (maximum complexity). The table presents both “lower bound” and “upper bound” scenarios to reflect the potential variability in complexity and thus potential needs. Overall, most diagnoses were categorized as “B”, with a variability observed in the category “D”, increasing from 49 adolescents in the “lower bound” scenario to 1,859 in the “upper bound” scenario. This wide range highlights the varying levels of care needed, with some adolescents requiring more intensive, multidisciplinary care. Copenhagen and Aarhus hospitals had higher numbers in the two categories “C” and “D” which include more complex diagnoses. The differences across hospitals may reflect variations in referral patterns or specialty distribution, suggesting potential inequities in tertiary-level transition readiness. S1 Fig and S5 Table presents the distribution of adolescents in the final pediatric surgical transition cohort, categorized by the degree of surgical transition needs across Danish tertiary hospitals.

**Table 1:** Distribution of unique adolescents per hospital, 16- to 17-year-old, categorized by disease complexity (A–D).

Disease complexity (A-D)	Overall (All Danish tertiary hospitals)		Copenhagen University Hospital Rigshospitalet		Aarhus University Hospital		Odense University Hospital		Aalborg University Hospital	
	*Lower bound*	*Upper* *bound*	*Lower bound*	*Upper bound*	*Lower bound*	*Upper bound*	*Lower bound*	*Upper bound*	*Lower bound*	*Upper bound*
**A**	2,041	1,191	567	296	491	263	638	423	383	231
**B**	3,232	2,324	1,096	822	1,021	740	819	540	384	284
**C**	1,450	1,398	566	510	381	393	399	390	150	154
**D**	49	1,859	24	625	8	505	10	514	7	255

Distribution of unique adolescents per hospital, aged 16–17, with disease complexity ranging from A (lowest) to D (highest). If an adolescent has multiple diagnoses requiring transitional care, they are classified in the most severe category applicable. The table presents two scenarios: The “lower bound” scenario assumes the least complex interpretation of each diagnosis, while the “upper bound” scenario assumes the highest possible complexity. Adolescents can meet the inclusion criteria for more than one hospital, so the total number of individuals does not sum up across hospitals.

### Variation

**Fig 3:**
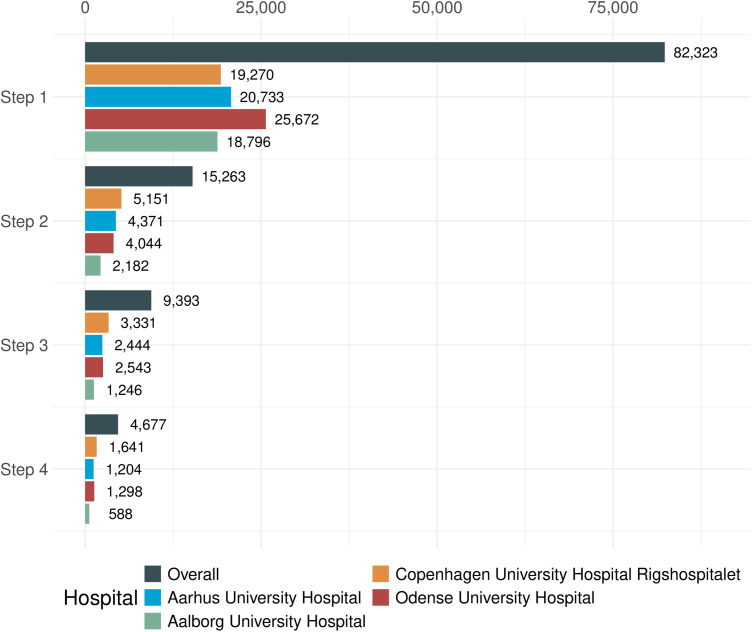
Representation of unique adolescents in each sub-cohort step 1-4 by hospital. The overall is visualized in the left-hand side of Fig 1. Note that it was possible for an adolescent to meet the inclusion criteria for more than one hospital, and thus the numbers do not amount to the overall total. The sub-cohort steps are defined as: Step 1 includes adolescents aged 12-17 years with one outpatient visit at a tertiary Danish hospital (2019-2022); Step 2 includes those with at least one outpatient visit related to a diagnosis associated with an expected need for comprehensive transitional care; Step 3 includes those with at least two outpatient visits related to a diagnosis associated with an expected need for comprehensive transitional care; and Step 4 includes those with two outpatient visits related to a diagnosis associated with an expected need for comprehensive transitional care, with at least one visit occurring between the ages of 16-17.

The absolute number of adolescents aged 12–17 with at least one outpatient visit during 2019–2022 was rather similar across the four tertiary hospitals as illustrated in Fig 3. Odense University Hospital received outpatient visits from 25,672 unique outpatients, whereas Aalborg University Hospital received 18,796. However, the proportion of outpatients with at least one diagnosis associated with an expected need for transfer to specialized adult care differed notably between hospitals, ranging from 11.6% at Aalborg University Hospital (2,182 out of 18.796) to 26.7% at Copenhagen University Hospital Rigshospitalet (5,151 out of 19,270).

### Supplementary analyses

An additional analysis was conducted using the pediatric department approach. The results of this approach are presented in the supporting material (S2 Fig). This approach resulted in the identification of 7,821 unique adolescents, a higher number than the 4,677 adolescents identified using the tertiary hospital approach. The overlap between both approaches, identified as 3,601 adolescents, is also detailed in the supplementary materials.

Finally, the supporting material also includes information on the most frequently occurring diagnoses overall and by hospital (presented in S6 Table), with, across all hospitals, juvenile arthritis and asthma being among the most common diagnoses identified. The information on diagnoses was summarized using the WHO-chapters I-XXI and the 3-digit ICD-10 codes.

## Discussion

### Interpretation

While we acknowledge that all adolescents with chronic conditions benefit from some level of transitional care, the approach presented in this study prioritized the identification of adolescents based on the complexity of their condition and the associated potential health risks, posed by inadequate transfer to adult care [33,34]. These risks include a higher likelihood of treatment failure, loss to follow-up, and an associated rise in morbidity and mortality [35,36]. Accordingly, our method was intentionally designed to be specific, aiming to capture individuals with the most intensive and complex needs, as a first step toward developing scalable tools for use at a population level. Applying this method also revealed notable differences in the proportion of adolescents identified across hospitals, ranging from 11.6% to 26.7%, suggesting that transition practices and referral patterns may vary.

It is also important to note that while chronic illnesses are more prevalent in younger children [19], our study population included only those adolescents who had outpatient visits at tertiary hospitals during their adolescent years. As such, the cohort did not include adolescents followed exclusively in primary or secondary care, or in specialties such as orthopedics, dermatology, ophthalmology, or otorhinolaryngology, where care transitions typically occur within the same department in Denmark. These design choices, combined with our methodological emphasis on diagnostic complexity, contribute to a lower yield compared to broader prevalence estimates reported in the literature.

To address this limitation, we included a supplementary analysis (S2 Fig) showing how the approach can be extended across all pediatric departments nationwide, nearly doubling the number of identified adolescents. Thus, while our estimates reflect a specific application, they demonstrate a scalable framework that can be adapted to different clinical settings and policy needs.

The methodology presented here is adaptable and may be useful for policymakers and health systems seeking to estimate the number of adolescents needing transition care at a national or regional level, facilitating more targeted healthcare planning.

### Comparison with the background literature

Previous research emphasizes that structured transitional care improves adherence to care, quality of life, and healthcare utilization. A review of 19 studies from 2016 to 2018 highlighted these welfare gains among adolescents with special health care needs [2]. Similarly, a review of 43 studies from 1995 to 2016 [37] found positive outcomes in population health and patient experience. These findings accentuate the importance of evaluating transitional care across domains.

A structured approach to transition planning benefits from a framework to identify adolescents who may require specialized adult healthcare. Mokkink et al. [13], proposed a comprehensive definition of chronic conditions in childhood, applicable to both somatic and psychiatric disorders, for use in epidemiological research and healthcare planning in the Netherlands. This definition involved a review of existing definitions and consultations with 27 experts. The final definition, applicable to children and adolescents aged 0–18 years, addresses a broad range of conditions, including both somatic and psychiatric disorders. In comparison with existing literature [15], our study’s narrower focus on complex conditions explains Denmark’s reported lower prevalence of transition needs, as it excludes individuals with less intensive care requirement.

Building on this, while some studies have focused on specific subspecialties like pediatric surgery, urology, and congenital heart disease [23–25], our study offers a broader, diagnosis-based framework applicable across multiple specialties. This framework is more suited for identifying adolescents in the general population who require comprehensive transitional care. By categorizing diagnoses based on disease complexity, we take into account that adolescents receive the appropriate level of care during their transition process. As complexity increases, so does the need for more intensive care, but due to limited resources, complexity categorization helps prioritize individuals with the highest needs, ultimately improving outcomes.

### Key findings and their implications

A priori, it was not possible to deduce whether the tertiary hospital or pediatric department approach would result in the largest population of adolescents in need of comprehensive transitional care, without detailed knowledge of the distribution of adolescents across hospitals and the visits at pediatric departments within hospitals. The tertiary hospital approach excluded all non-tertiary hospitals, but all types of departments were included, whereas the pediatric department approach was nationwide, but only included pediatric departments.

Prior to the initiation of the present manuscript, another two approaches attempting to identify adolescents in need of comprehensive transitional care were assessed. The first of these preliminary approaches was based on identifying adolescents with at least two outpatient visits per year within two consecutive calendar years. The second preliminary approach involved identifying adolescents who had attended more than five outpatient visits within a single calendar year. However, these preliminary approaches were deemed too unspecific in terms of identifying adolescents with an expected need for comprehensive transitional care. The methods captured adolescents with diagnoses that were not indicative of long-term conditions, such as a large number of outpatient visits with diagnoses pertaining to suspected disease or observation (from ICD-10 chapter XXI, i.e., Z-codes), and adolescents seen for standard orthopedic procedures. The complexity of the final approach reflects its ability to more accurately identify adolescents most likely to benefit from structured transitional care, distinguishing them from those with less complex conditions or non-chronic health issues.

A reduction in the population was observed when moving from sub-cohort step 1 to step 2; from all outpatient visits to those with diagnoses associated with an expected need of transfer to specialized adult care (S4 Table). Notably, 30% of excluded cases are ICD-coded with Z01, an encounter for general examination without complaint. This high frequency of Z01 diagnoses is calling for more specific diagnostic codes and warranting further investigation into coding practices.

Some adolescents only turned 12 during the latter part of the study period (2019–2022). These are part of the 12–17 age group, but not the 16–17 age group, highlighting varying person-time available for outpatient visits while in these age groups. Similarly, those aged 17 at the start of 2019 had less than a year to meet the outpatient visit criteria. For adolescents with surgical diagnoses (presented in S2 Table), who transferred to adult care at age 16 in Denmark, the relevant age groups are 12–15 for the background population and 14–15 for inclusion in the final surgical study cohort. For this cohort, the prevalence of lower severity of complexity at Odense and Aalborg University Hospital was expected, as Aarhus University Hospital and Copenhagen University Hospital Rigshospitalet are center for complex surgical diseases in Denmark. The variation in complexity across hospitals and the differences in surgical care needs highlight the importance of understanding regional differences in healthcare delivery when planning for transitional care.

Lastly, it should be mentioned, that although the data collection period (2019−2022) overlapped with the COVID-19 pandemic, it did not lead to a decrease in tertiary hospital contacts among 16- to 17-year-old adolescents transferring from pediatric to adult healthcare in Denmark (Fig 2), or 14- to 15-year-old in the surgical transition cohort (S3 Fig). These results indicate that the isolation measures and other pandemic-related restrictions did not impact the number of cases or the continuity of care at tertiary hospitals. This observation also underlines the high specificity of the suggested approach. The resilience of healthcare transitions during the pandemic suggests that the framework used here could be effective in maintaining continuity of care during other potential disruptions.

### Strengths and limitations

The study has several strengths. It is based on a nationwide, population-based cohort using comprehensive Danish health registers, ensuring complete follow-up and high internal validity. The involvement of clinicians from multiple subspecialties strengthened the diagnostic classification by grounding it in clinical expertise.

The methodology and results outlined here are intended as “proof of concept” to provide a preliminary understanding of complexity and thus transitional care needs. Thus, it should be seen as a foundation for future work rather than a definitive guide. Defining the need for transitional care solely based on somatic diagnoses is a limited approach, as many adolescents have multiple diagnoses, including psychiatric conditions, which were not included in this study but may contribute to increased complexity and care needs. Additionally, a comprehensive assessment requires more than just diagnostic codes, as these may contain errors or lack sufficient detail to fully capture an adolescent’s transitional care requirements. Furthermore, psychosocial, socioeconomic, and developmental factors, as well as healthcare access and resource availability, also influence transition needs but were not incorporated into this framework. Future research should aim to integrate these dimensions for a more holistic approach.

One conceptual limitation is that the categorization of adolescents into different degrees of transition needs is somewhat subjective, relying on clinical expert evaluation. In this study, all specialists involved were from Copenhagen University Hospital Rigshospitalet, which may influence categorization due to localized practices or perspectives. Although classifications were developed within a single tertiary center, the involvement of multiple subspecialties increases the representativeness of the categorization. Nevertheless, future multicenter validation would help reduce potential local bias.

Additionally, a practical limitation is that some adolescents are followed in the primary care sector, where diagnoses are not reported to the DNPR, making it difficult to determine the exact number. However, in Denmark, relatively few children and adolescents with complex chronic illnesses receive their primary follow-up care outside the hospital system.

Finally, the broad categorization used in Table 1 introduces a degree of uncertainty, as the gap between the lower and upper bound scenarios is substantial. This variability makes it challenging to draw precise conclusions regarding the exact number of adolescents within each complexity category.

## Conclusion

This study provides a methodological and reproducible framework for identifying adolescents with the greatest need for comprehensive transitional care within specialized somatic healthcare. By applying diagnosis-based criteria and expert-informed complexity levels to national health register data, the approach offers an initial, systematic means of estimating transition needs at a population level. While the framework serves as an important first step toward understanding the distribution and intensity of transitional care needs, it does not yet account for the full range of factors influencing transition readiness. Future research should extend this approach to incorporate psychiatric, socio-economic, and developmental determinants, moving toward a more comprehensible and equitable model for assessing readiness for the transition to adult healthcare.

## Supporting information

S1 Table Representation of criteria when selection outpatients from the Danish National Patient Register. Additionally, the contacts were restricted to a maximum duration of one hour. This was achieved by calculating the contact duration, defined as the difference between the start time and end time for a contact. It was assumed that elective contacts would not span two consecutive dates, thereby not being scheduled to commence between 11 PM and midnight. Visits with a duration of zero were excluded, as it could not be determined why these visits ended at the same time as they started. Variable is given as the Danish variable name in the Danish National Patient Register, and Value(s) the observation related to outpatient visits.
(DOCX)

S2 Table Representation of diagnosis where a degree of transition needs from pediatric to adult health care is expected. The degree of transition need reflected in disease complexity is categorized A-D, with A being minimal transition health care need, and D being maximum transition health care need. The degree of transition need for each diagnosis was categorized by consulting clinical experts. Additionally, surgeons were consulted to provide specialized insights into pediatric surgical diagnoses that may require health care transition, here denoted S.
(DOCX)

S1 Fig Flowchart illustrating the number of unique individuals in each of the surgical cohort steps. The left hand describes the distribution of individuals in each step. The right-hand side of the figure is included to acknowledge the fact that not all individuals in the cohort reach age 14–15 years during the study period.
(TIFF)

S3 Table List of hospitals in Denmark with a pediatric department. Each department is presented with a hospital code and department code for identification in the Danish National Patient register.
(DOCX)

S2 Fig Flowchart illustrating the number of unique adolescents in each of the pediatric sub-cohort steps. The left hand describes the distribution of adolescents in each step. The right-hand side of the figure is included to acknowledge the fact that not all adolescents in the pediatric-department cohort will reach age 16–17 years during the study period.
(TIFF)

S3 Fig Distribution of adolescents, 14- to 15-year-old, with outpatient visits for surgical transitional care need across hospitals in each calendar year. No decrease in the number of adolescents was observed during the COVID-19 pandemic from 2020 to 2021. Note that the individuals may be associated with multiple hospitals, so the totals do not sum across hospitals.
(TIF)

S4 Table Diagnoses associated with exclusion from the final study population. Most common primary diagnosis (3-digit ICD-10 codes) per visit for individuals included in specificity step 1 (Individuals aged 12–17 years with at least one outpatient visit at a tertiary Danish hospital during 2019–2022) and excluded in step 2 (Individuals aged 12–17 years with at least one outpatient visit at a tertiary Danish hospital with a diagnosis associated with an expected need for transitional care during 2019–2022), as the diagnose related to the tertiary hospital visit is not qualified as a need of transition diagnosis. N is the number of visits with the diagnosis listed, and the total number of outpatient visits is 177,583.
(DOCX)

S5 Table Distribution of unique adolescents per hospital, 14- to 15-year-old, in the categorization of degree of complexity for surgical diagnosis. Complexity is categorized as severity A to D, with A being minimum expected need for transition, and D maximum. If an individual has more than one diagnosis requiring transition of care, they are included in the most severe group they qualify for. Two scenarios are represented, “lower bound” scenarios refer to the minimal expected need of transition care applied to a diagnosis, “upper bound” refers to the maximum. Individuals are represented as unique per hospital, and 14–15 years of age by the time of the hospital contact.
(DOCX)

S6 Table The top ten transition diagnoses for the tertiary population for all individuals across tertiary hospitals. Representation of the ten most common transition diagnoses (3-digit ICD-10 codes) for individuals with at least two visits at tertiary hospitals with diagnoses associated with an expected need for transitional care while aged 12–17 years, with at least one visit at age 16- to17-years -old. Numbers (N) are unique individuals with an outpatient visit with the diagnosis, percentages relative to the overall number of unique individuals.
(DOCX)
