# Supplementary material for: Defining the population of adolescents in need of comprehensive transitional care based on diagnosis, visit frequency, and disease complexity
Source: PLoS One. 2026 Jan 27;21(1):e0339721. doi: 10.1371/journal.pone.0339721 (PMC12843535; doi:10.1371/journal.pone.0339721)
Supplement: S1 Table — Additionally, the contacts were restricted to a maximum duration of one hour. This was achieved by calculating the contact duration, defined as the difference between the start time and end time for a contact. It was assumed that elective contacts would not span two consecutive dates, thereby not being scheduled to commence between 11 PM and midnight. Visits with a duration of zero were excluded, as it could not be determined why these visits ended at the same time as they started. Variable is given as the Danish variable name in the Danish National Patient Register, and Value(s) the observation related to outpatient visits. (DOCX) [file pone.0339721.s001.docx]

**S1 Table. Representation of criteria when selection outpatients from the Danish National Patient Register**.

| **Variable** | **Value(s)** | **Explanation** |
| --- | --- | --- |
| prioritet | “ATA3” | Planned visits (neither acute nor sub-acute) |
| kontaktaarsag | “Ukendt”, “ALCC01”, “ALCC90” | Reason behind contact either unknown, disease, or other, since it is not mandatory to register this information for planned/elective contacts |
| kontakttype | “ALCA00” | Physical contact (e.g., not virtual consultations) |

Additionally, the contacts were restricted to a maximum duration of one hour. This was achieved by calculating the contact duration, defined as the difference between the start time and end time for a contact. It was assumed that elective contacts would not span two consecutive dates, thereby not being scheduled to commence between 11 PM and midnight. Visits with a duration of zero were excluded, as it could not be determined why these visits ended at the same time as they started. Variable is given as the Danish variable name in the Danish National Patient Register, and Value(s) the observation related to outpatient visits.
