# Supplementary material for: Defining the population of adolescents in need of comprehensive transitional care based on diagnosis, visit frequency, and disease complexity
Source: PLoS One. 2026 Jan 27;21(1):e0339721. doi: 10.1371/journal.pone.0339721 (PMC12843535; doi:10.1371/journal.pone.0339721)
Supplement: S2 Table — The degree of transition need reflected in disease complexity is categorized A-D, with A being minimal transition health care need, and D being maximum transition health care need. The degree of transition need for each diagnosis was categorized by consulting clinical experts. Additionally, surgeons were consulted to provide specialized insights into pediatric surgical diagnoses that may require health care transition, here denoted S. (DOCX) [file pone.0339721.s002.docx]

**S2 Table. Representation of diagnosis where a degree of transition needs from pediatric to adult health care is expected**.

| **ICD-10** | **Disease specification** | **Complexity,**  **A-D** | **Surgical,**  **S** |
| --- | --- | --- | --- |
| **I. Certain infectious and parasitic diseases** | | | |
| A689 | Relapsing fever, unspecified | A |  |
| B18 | Chronic viral hepatitis | A |  |
| B20-B24 | Human immunodeficiency virus [HIV] disease | B |  |
| **II. Neoplasms** | | | |
| C01 | Malignant neoplasm of base of tongue | B-C |  |
| C03 | Malignant neoplasm of gum | B-C |  |
| C04 | Malignant neoplasm of floor of mouth | B-C |  |
| C05 | Malignant neoplasm of palate | B-C |  |
| C07 | Malignant neoplasm of parotid gland | B-C |  |
| C10 | Malignant neoplasm of oropharynx | B-C |  |
| C11 | Malignant neoplasm of nasopharynx | B-C |  |
| C15 | Malignant neoplasm of esophagus | B-C |  |
| C20 | Malignant neoplasm of rectum | B-C |  |
| C22 | Malignant neoplasm of liver and intrahepatic bile ducts | B-C |  |
| C25 | Malignant neoplasm of pancreas | B-C |  |
| C30 | Malignant neoplasm of nasal cavity and middle ear | B-C |  |
| C31 | Malignant neoplasm of accessory sinuses | B-C |  |
| C40 | Malignant neoplasm of bone and articular cartilage of limbs | B-C |  |
| C41 | Malignant neoplasm of bone and articular cartilage of other and unspecified sites | B-C |  |
| C43 | Malignant melanoma of skin | B-C |  |
| C44 | Other malignant neoplasms of skin | B-C |  |
| C47 | Malignant neoplasm of peripheral nerves and autonomic nervous system | B-C |  |
| C48 | Malignant neoplasm of retroperitoneum and peritoneum | B-C |  |
| C49 | Malignant neoplasm of other connective and soft tissue | B-C |  |
| C52 | Malignant neoplasm of vagina | B-C |  |
| C54 | Malignant neoplasm of corpus uteri | B-C |  |
| C56 | Malignant neoplasm of ovary | B-C |  |
| C61 | Malignant neoplasm of prostate | B-C |  |
| C62 | Malignant neoplasm of testis | B-C |  |
| C64 | Malignant neoplasm of kidney, except renal pelvis | B-C |  |
| C65 | Malignant neoplasm of renal pelvis | B-C |  |
| C66 | Malignant neoplasm of ureter | B-C |  |
| C67 | Malignant neoplasm of bladder | C |  |
| C68 | Malignant neoplasm of other and unspecified urinary organs | B-C |  |
| C69 | Malignant neoplasm of eye and adnexa | C |  |
| C70 | Malignant neoplasm of meninges | C-D |  |
| C71 | Malignant neoplasm of brain | C-D |  |
| C72 | Malignant neoplasm of spinal cord, cranial nerves, and other parts of central nervous system | C-D |  |
| C73 | Malignant neoplasm of thyroid gland | C |  |
| C74 | Malignant neoplasm of adrenal gland | C |  |
| C81-C96 | Malignant neoplasms, stated or presumed to be primary, of lymphoid, hematopoietic, and related tissue | B-C |  |
| D00-D09 | In situ neoplasms | B-C |  |
| D16 | Benign neoplasm of bone and articular cartilage | B |  |
| D18 | Hemangioma and lymphangioma, any site | B |  |
| D21 | Other benign neoplasms of connective and other soft tissue | B |  |
| D23 | Other benign neoplasms of skin | B |  |
| D32 | Benign neoplasm of meninges | C-D |  |
| D33 | Benign neoplasm of brain and other parts of central nervous system | C-D |  |
| D39 | Neoplasm of uncertain or unknown behavior of female genital organs | B-C |  |
| D40 | Neoplasm of uncertain or unknown behavior of male genital organs | B-C |  |
| D41 | Neoplasm of uncertain or unknown behavior of urinary organs | B-C |  |
| D42 | Neoplasm of uncertain or unknown behavior of meninges | C-D |  |
| D43 | Neoplasm of uncertain or unknown behavior of brain and central nervous system | C-D |  |
| D46 | Myelodysplastic syndromes | C |  |
| D469B | Refractory neutropenia | B-C |  |
| **III. Diseases of the blood and blood-forming organs and certain disorders involving the immune mechanism** | | | |
| D55-D59 | Hemolytic anemia | B-C |  |
| D60 | Acquired pure red cell aplasia (erythroblastopenia) | B-C |  |
| D61 | Other aplastic anemias | C |  |
| D669E | Hemophilia A severe | B |  |
| D679D | Hemophilia B severe | B |  |
| D680 | Von Willebrand disease | B-C |  |
| D683 | APL | B |  |
| D70 | Neutropenia | B-C |  |
| D709 | Neutropenia unspecified | B-C |  |
| D719C | Chronic granulomatous disease in children | C-D |  |
| D762 | Hemophagocytic syndrome, infection-associated | C |  |
| D800 | Hereditary hypogammaglobulinemia | C |  |
| D801 | Nonfamilial hypogammaglobulinemia | C |  |
| D802 | Selective deficiency of immunoglobulin A [IgA] | A |  |
| D803 | Selective deficiency of immunoglobulin G [IgG] subclasses | C |  |
| D804 | Selective deficiency of immunoglobulin M [IgM] | C |  |
| D805 | Immunodeficiency with increased immunoglobulin M [IgM] | C-D |  |
| D806 | Antibody deficiency with near-normal immunoglobulins or with hyperimmunoglobulinaemia | B-D |  |
| D807 | Transient hypogammaglobulinemia of infancy | A |  |
| D808 | Other immunodeficiencies with predominantly antibody defects | C |  |
| D809 | Immunodeficiency with predominantly antibody defects, unspecified | C |  |
| D81 | Combined immunodeficiencies | C-D |  |
| D82 | Immunodeficiency associated with other major defects | C-D |  |
| D824 | Hyperimmunoglobulin E (IgE) syndrome | C |  |
| D828 | Immunodeficiency associated with other specified major defects | C |  |
| D829 | Immunodeficiency associated with major defect, unspecified | C |  |
| D83 | Common variable immunodeficiency | C |  |
| D831 | Common variable immunodeficiency with predominant immunoregulatory T-cell disorders | C-D |  |
| D84 | Other immunodeficiencies | B-C |  |
| D840 | Lymphocyte function antigen-1 [LFA-1] defect | C-D |  |
| D848 | Other specified immunodeficiencies | A-C |  |
| D849 | Immunodeficiency, unspecified | A-C |  |
| D86 | Sarcoidosis | B |  |
| D891 | Cryoglobulinemia | B |  |
| **IV. Endocrine, nutritional, and metabolic diseases** | | | |
| E00 | Congenital iodine-deficiency syndrome | C |  |
| E03 | Other hypothyroidism | A |  |
| E05 | Thyrotoxicosis [hyperthyroidism] | B |  |
| E10-E14 | Diabetes mellitus | B |  |
| E20 | Hypoparathyroidism | B |  |
| E21 | Hyperparathyroidism and other disorders of parathyroid gland | B |  |
| E22 | Hyperfunction of pituitary gland | B |  |
| E23 | Hypofunction and other disorders of pituitary gland | C |  |
| E24 | Cushing syndrome | B |  |
| E25 | Adrenogenital disorders | C |  |
| E26 | Hyperaldosteronism | B |  |
| E271 | Primary adrenocortical insufficiency | B |  |
| E28 | Ovarian dysfunction | B |  |
| E29 | Testicular dysfunction | B |  |
| E31 | Polyglandular dysfunction | B |  |
| E70 | Disorders of aromatic amino-acid metabolism | A-C |  |
| E71 | Disorders of branched-chain amino-acid metabolism and fatty-acid metabolism | A-D |  |
| E72 | Cystinosis | A-C |  |
| E73 | Lactose intolerance | A |  |
| E74 | Other disorders of carbohydrate metabolism | B-C |  |
| E75 | Disorders of sphingolipid metabolism and other lipid storage disorders | C-D |  |
| E76 | Disorders of glycosaminoglycan metabolism | C-D |  |
| E77 | Disorders of glycoprotein metabolism | C |  |
| E78 | Disorders of lipoprotein metabolism and other lipidaemia | A |  |
| E79 | Disorders of purine or pyrimidine metabolism | A |  |
| E80 | Disorders of porphyrin and bilirubin metabolism | B |  |
| E83 | Disorder of mineral metabolism | A |  |
| E84 | Cystic fibrosis | B |  |
| E85 | Amyloidosis | B |  |
| E880A | Alfa-1-antitrypsinmangel | A-B |  |
| **V. Mental and behavioral disorders** | | | |
| F842 | Rett syndrome | C-D |  |
| **VI. Diseases of the nervous system** | | | |
| G04 | Encephalitis, myelitis, and encephalomyelitis | A-D |  |
| G09 | Sequelae of inflammatory diseases of central nervous system | D |  |
| G11 | Hereditary ataxia | D |  |
| G113 | Cerebellar ataxia with defective DNA repair | C-D |  |
| G12 | Spinal muscular atrophy and related syndromes | B-D |  |
| G24 | Dystonia | D |  |
| G31 | Other degenerative diseases of nervous system, not elsewhere classified | C-D |  |
| G35 | Multiple sclerosis | C-D |  |
| G37 | Other demyelinating diseases of central nervous system | C-D |  |
| G40 | Epilepsy | A-D |  |
| G60 | Hereditary and idiopathic neuropathy | C-D |  |
| G70 | Myasthenia gravis and other myoneural disorders | D |  |
| G71 | Primary disorders of muscles | C-D |  |
| G72 | Other myopathies | D |  |
| G736 | Myopathy in metabolic diseases | D |  |
| G80-G83 | Cerebral palsy and other paralytic syndromes | C-D |  |
| G91 | Hydrocephalus | B-D |  |
| G92 | Toxic encephalopathy | C-D |  |
| G931 | Anoxic brain damage, not elsewhere classified | C-D |  |
| G941 | Hydrocephalus in neoplastic disease | C-D |  |
| G958C | Neurogenic bladder in spinal cord injury | C | S |
| **IX. Diseases of the circulatory system** | | | |
| I009 | Rheumatic fever without heart disease | A |  |
| I05-I09 | Chronic rheumatic heart diseases | A |  |
| I11 | Hypertensive heart disease | B |  |
| I12 | Hypertensive renal disease | B |  |
| I13 | Hypertensive heart and renal disease | C |  |
| I27 | Other pulmonary heart diseases | B |  |
| I34 | Nonrheumatic mitral valve disorders | A |  |
| I350 | Aortic (valve) stenosis | A-C |  |
| I36 | Nonrheumatic tricuspid valve disorders | A |  |
| I37 | Pulmonary valve disorders | A |  |
| I42 | Cardiomyopathy | B-C |  |
| I442 | Atrioventricular block, complete | A |  |
| I50 | Heart failure | C |  |
| I63 | Cerebral infarction | B-D |  |
| I677 | Vasculitis CNS | C |  |
| I819 | Portal vein thrombosis | B |  |
| **X. Diseases of the respiratory system** | | | |
| J43 | Emphysema | B |  |
| J448 | Other specified chronic obstructive pulmonary disease | B |  |
| J449 | Chronic obstructive pulmonary disease, unspecified | B |  |
| J45 | Asthma | A |  |
| J47 | Bronchiectasis | B |  |
| J84 | Other interstitial pulmonary diseases | B |  |
| J953 | Chronic pulmonary insufficiency following surgery | C |  |
| J961 | Chronic respiratory failure | C-D |  |
| **XI. Diseases of the digestive system** | | | |
| K50 | Crohn disease [regional enteritis] | B-C |  |
| K51 | Ulcerative colitis | B-C |  |
| K592 | Neurogenic bowel, not elsewhere classified | C | S |
| K73 | Chronic hepatitis, not elsewhere classified | B |  |
| K74 | Fibrosis and cirrhosis of liver | C |  |
| K75 | Other inflammatory liver diseases | B |  |
| K76 | Other diseases of liver | C |  |
| K830 | Cholangitis | B |  |
| K86 | Other chronic pancreas | B |  |
| K90 | Intestinal malabsorption | A-D |  |
| K912 | Postsurgical malabsorption, not elsewhere classified | B-D | S |
| K912B | Short bowel syndrome | C | S |
| **XII. Diseases of the skin and subcutaneous tissue** | | | |
| L928G | Chronic granulomatous disease | C-D |  |
| **XII. Diseases of the musculoskeletal system and connective tissue** | | | |
| M029 | Reactive arthropathy, unspecified | A |  |
| M076 | Other enteropathic arthropathies | B |  |
| M080A | Juvenile rheumatoid arthritis RF pos | B |  |
| M080B | Juvenile rheumatoid arthritis RF neg | A |  |
| M082 | Juvenile arthritis with systemic onset | B-C |  |
| M083 | Juvenile polyarthritis (seronegative) | B |  |
| M084 | Pauciarticular juvenile arthritis | B |  |
| M088A | Enthesitis-related arthritis | B |  |
| M089 | Juvenile arthritis, unspecified | B |  |
| M090 | Juvenile arthritis in psoriasis | B |  |
| M091 | Juvenile arthritis in Crohn disease [regional enteritis] | C |  |
| M092 | Juvenile arthritis in ulcerative colitis | C |  |
| M123 | Palindromic rheumatism | A |  |
| M148 | Arthropathies in other specified diseases classified elsewhere | C |  |
| M313 | Wegener granulomatosis | C |  |
| M314 | Aortic arch syndrome [Takayasu] | B |  |
| M320 | Drug-induced systemic lupus erythematosus | B |  |
| M329 | Systemic lupus erythematosus, unspecified | B-C |  |
| M332 | Polymyositis | B |  |
| M339 | Dermatopolymyositis, unspecified | B |  |
| M348 | Other forms of systemic sclerosis | C |  |
| M349 | Systemic sclerosis, unspecified | C |  |
| M350 | Sicca syndrome [Sjögren] | C |  |
| M351A | Mixed connective tissue disease | B |  |
| M352 | Behçet disease | C |  |
| M354 | Diffuse (eosinophilic) fasciitis | B |  |
| M659 | Synovitis or tenosynovitis, unspecified | A |  |
| M722 | Plantar aponeuroses contracture | B |  |
| M863 | Chronic multifocal osteomyelitis | A |  |
| M941 | Relapsing polychondritis | B |  |
| **XIV. Diseases of the genitourinary system** | | | |
| N03 | Chronic nephritic syndrome | B |  |
| N04 | Nephrotic syndrome | B |  |
| N05 | Unspecified nephritic syndrome | B |  |
| N07 | Hereditary nephropathy, not elsewhere classified | B-C |  |
| N11 | Chronic tubulo-interstitial nephritis | B |  |
| N12 | Tubulo-interstitial nephritis, not specified as acute or chronic | B |  |
| N13 | Obstructive and reflux uropathy | A-B | S |
| N14 | Drug- and heavy-metal-induced tubulo-interstitial and tubular conditions | A |  |
| N182 | Chronic kidney disease, stage 2 | A |  |
| N183-N185 | Chronic kidney disease, stage 3-5 | C |  |
| N25 | Disorders resulting from impaired renal tubular function | B |  |
| N311 | Reflex neuropathic bladder, not elsewhere classified | C | S |
| N312 | Flaccid neuropathic bladder, not elsewhere classified | C | S |
| N35 | Urethral stricture | B | S |
| N39 | Other disorders of urinary system | B | S |
| N895 | Stricture and atresia of vagina | B | S |
| **XVI. Certain conditions originating in the perinatal period** | | | |
| P27 | Chronic respiratory disease originating in the perinatal period | B-C |  |
| **XVII. Congenital malformations, deformations, and chromosomal abnormalities** | | | |
| Q00 | Anencephaly and similar malformations | B-D |  |
| Q01 | Encephalocele | B-D |  |
| Q02 | Microcephaly | B-D |  |
| Q03 | Congenital hydrocephalus | B-D |  |
| Q04 | Other congenital malformations of brain | B-D |  |
| Q05 | Spina bifida | B-D | S |
| Q20 | Congenital malformations of cardiac chambers and connections | B |  |
| Q210 | Ventricular septal defect | A |  |
| Q211 | Atrial septal defect | A |  |
| Q212 | Atrioventricular septal defect | B |  |
| Q213 | Tetralogy of Fallot | B |  |
| Q221 | Congenital pulmonary valve stenosis | B |  |
| Q226 | Hypoplastic right heart syndrome | B-C |  |
| Q234 | Hypoplastic left heart syndrome | B-C |  |
| Q238 | Other congenital malformations of aortic and mitral valves | A |  |
| Q24 | Other congenital malformations of heart | A-D |  |
| Q25 | Congenital malformations of great arteries. Q250 not included. | A-B |  |
| Q252 | Atresia of aorta | B |  |
| Q26 | Congenital malformations of great veins | A-B |  |
| Q262 | Total anomalous pulmonary venous connection | B |  |
| Q39 | Congenital malformations of oesophagus | B-C | S |
| Q41 | Congenital absence, atresia and stenosis of small intestine | B | S |
| Q42 | Congenital absence, atresia and stenosis of large intestine | A-D | S |
| Q431 | Hirschsprung disease | B | S |
| Q44 | Congenital malformations of gallbladder, bile ducts and liver | B | S |
| Q447 | Other congenital malformations of liver | A-B | S |
| Q45 | Other congenital malformations of digestive system | B | S |
| Q50-Q52 | Congenital malformations of genital organs (except hypospadias, balanic). | B | S |
| Q53 | Undescended testicle | A | S |
| Q54-Q55 | Congenital malformations of genital organs (except hypospadias, balanic). Q540 not included | B | S |
| Q56 | Indeterminate sex and pseudohermaphroditism | C | S |
| Q601 | Renal agenesis, bilateral | C |  |
| Q611 | Polycystic kidney, autosomal recessive | C | S |
| Q612 | Polycystic kidney, autosomal dominant | A-B | S |
| Q613 | Polycystic kidney, unspecified | A-B | S |
| Q62 | Congenital obstructive defects of renal pelvis and congenital malformations of ureter | A |  |
| Q63 | Other congenital malformations of kidney | A-B | S |
| Q64 | Other congenital malformations of urinary system | A-B | S |
| Q71 | Deficient development of upper extremity | A |  |
| Q72 | Deficient development of lower extremity | A |  |
| Q74 | Other congenital malformations of limb(s) | A |  |
| Q75 | Other congenital malformations of skull and face bones | A-B |  |
| Q76 | Congenital malformations of spine and bony thorax | B |  |
| Q77 | Osteochondrodysplasia with defects of growth of tubular bones and spine | C |  |
| Q78 | Other osteochondrodysplasias | A-C |  |
| Q79 | Congenital malformations of the musculoskeletal system, not elsewhere classified | B | S |
| Q796 | Ehlers-Danlos syndrome | C | S |
| Q822 | Mastocytosis | C-D |  |
| Q85 | Phacomatoses, not elsewhere classified | B-D |  |
| Q86 | Congenital malformation syndromes due to known exogenous causes, not elsewhere classified | C |  |
| Q87 | Other specified congenital malformation syndromes affecting multiple systems | C-D | S |
| Q89 | Other congenital malformations, not elsewhere classified | A-D |  |
| Q90 | Down syndrome | C-D |  |
| Q91 | Edwards syndrome and Patau syndrome | C-D |  |
| Q92 | Other trisomies and partial trisomies of the autosomes, not elsewhere classified | C-D |  |
| Q93 | Monosomies and deletions from the autosomes, not elsewhere classified | C-D |  |
| Q95 | Balanced rearrangements and structural markers, not elsewhere classified | B-D |  |
| Q96 | Turner syndrome | C |  |
| Q97 | Other sex chromosome abnormalities, female phenotype, not elsewhere classified | C |  |
| Q98 | Other sex chromosome abnormalities, male phenotype, not elsewhere classified | C |  |
| Q99 | Other chromosome abnormalities, not elsewhere classified | C |  |
| **XVIII. Symptoms, signs, and abnormal clinical and laboratory findings, not elsewhere classified** | | | |
| R15 | Fecal incontinence | B | S |
| R32 | Unspecified urinary incontinence | B | S |
| R620 | Delayed milestone | A-D |  |
| **XIX. Injury, poisoning and certain other consequences of external causes** | | | |
| T86 | Failure and rejection of transplanted organs and tissues | C |  |
| **XXI. Factors influencing health status and contact with health services** | | | |
| Z94 | Transplanted organ and tissue status (except skin transplant status). Z945 not included. | C | S |
| Z948C1 | Bone marrow transplanted with allogeneic bone marrow | C-D |  |

The degree of transition need reflected in disease complexity, is categorized A-D, with A being minimal transition health care need, and D maximum transition health care need. The degree of transition need for each diagnosis was categorized by consulting clinical experts. Additionally, surgeons were consulted to provide specialized insights into pediatric surgical diagnoses that may require health care transition, here denoted S.
