# Supplementary figures and images for: Defining the population of adolescents in need of comprehensive transitional care based on diagnosis, visit frequency, and disease complexity
Source: PLoS One. 2026 Jan 27;21(1):e0339721. doi: 10.1371/journal.pone.0339721 (PMC12843535; doi:10.1371/journal.pone.0339721)

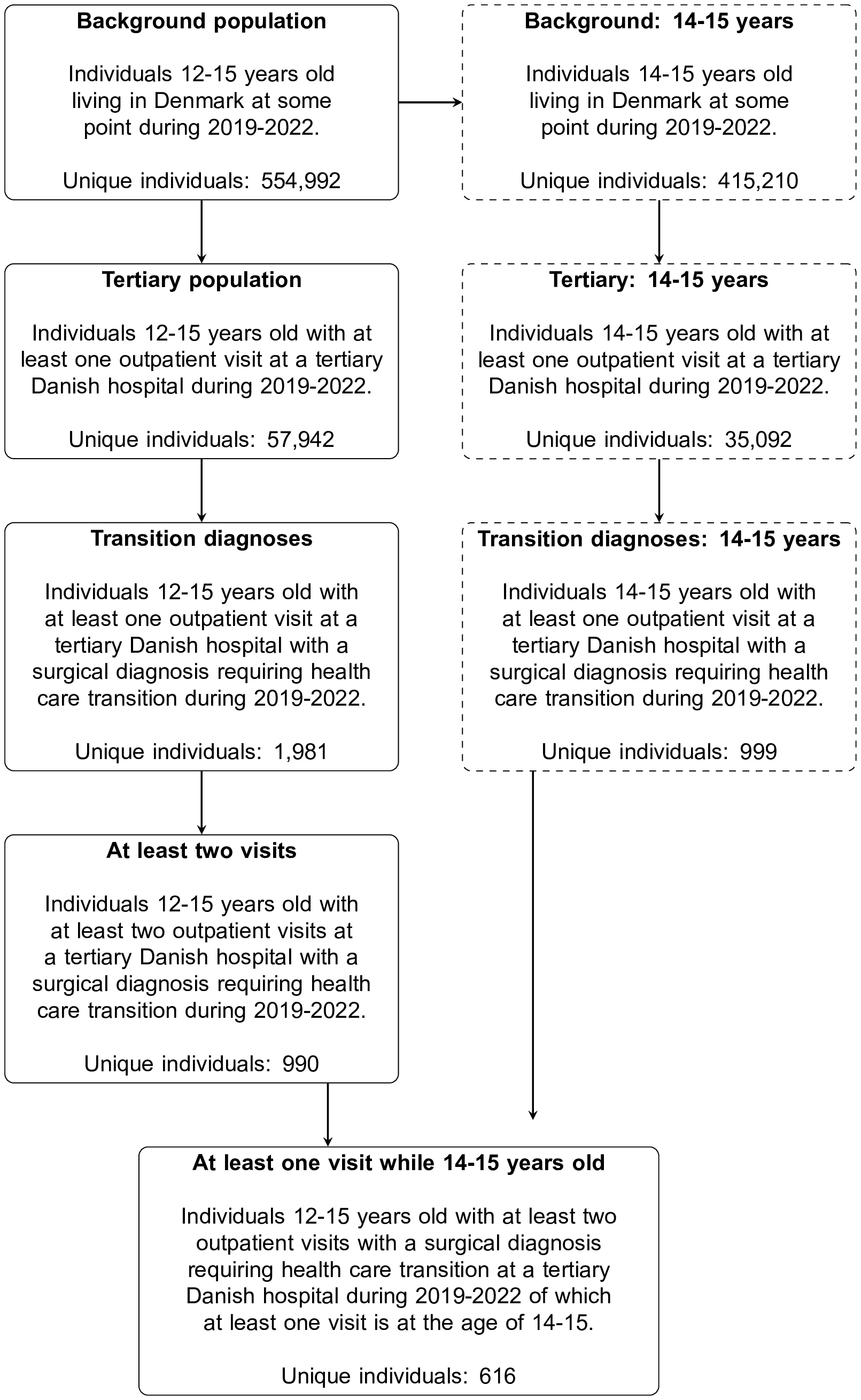

Supplement: S1 Fig — The left hand describes the distribution of individuals in each step. The right-hand side of the figure is included to acknowledge the fact that not all individuals in the cohort reach age 14–15 years during the study period. (TIFF) [file pone.0339721.s003.tiff]

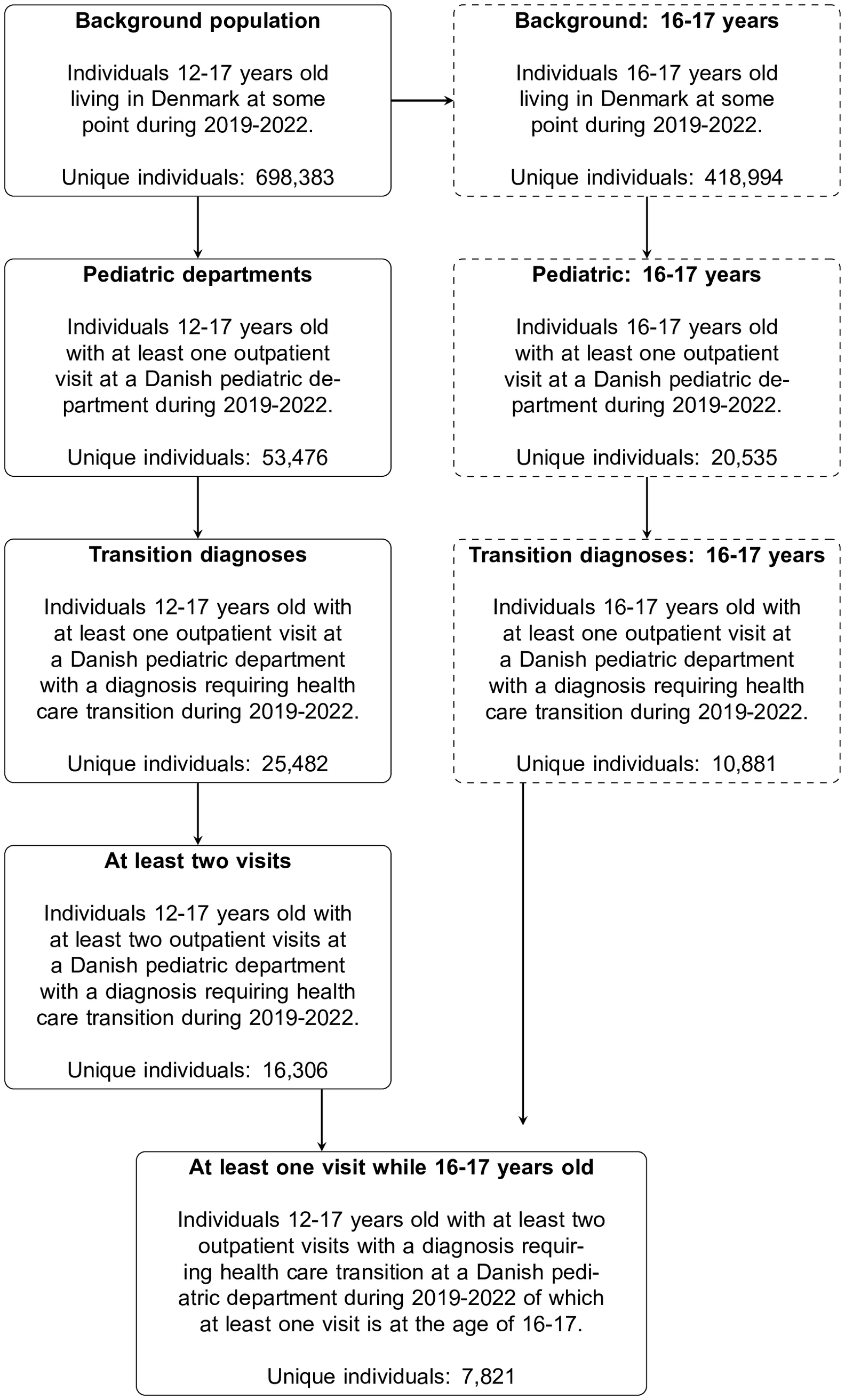

Supplement: S2 Fig — The left hand describes the distribution of adolescents in each step. The right-hand side of the figure is included to acknowledge the fact that not all adolescents in the pediatric-department cohort will reach age 16–17 years during the study period. (TIFF) [file pone.0339721.s005.tiff]

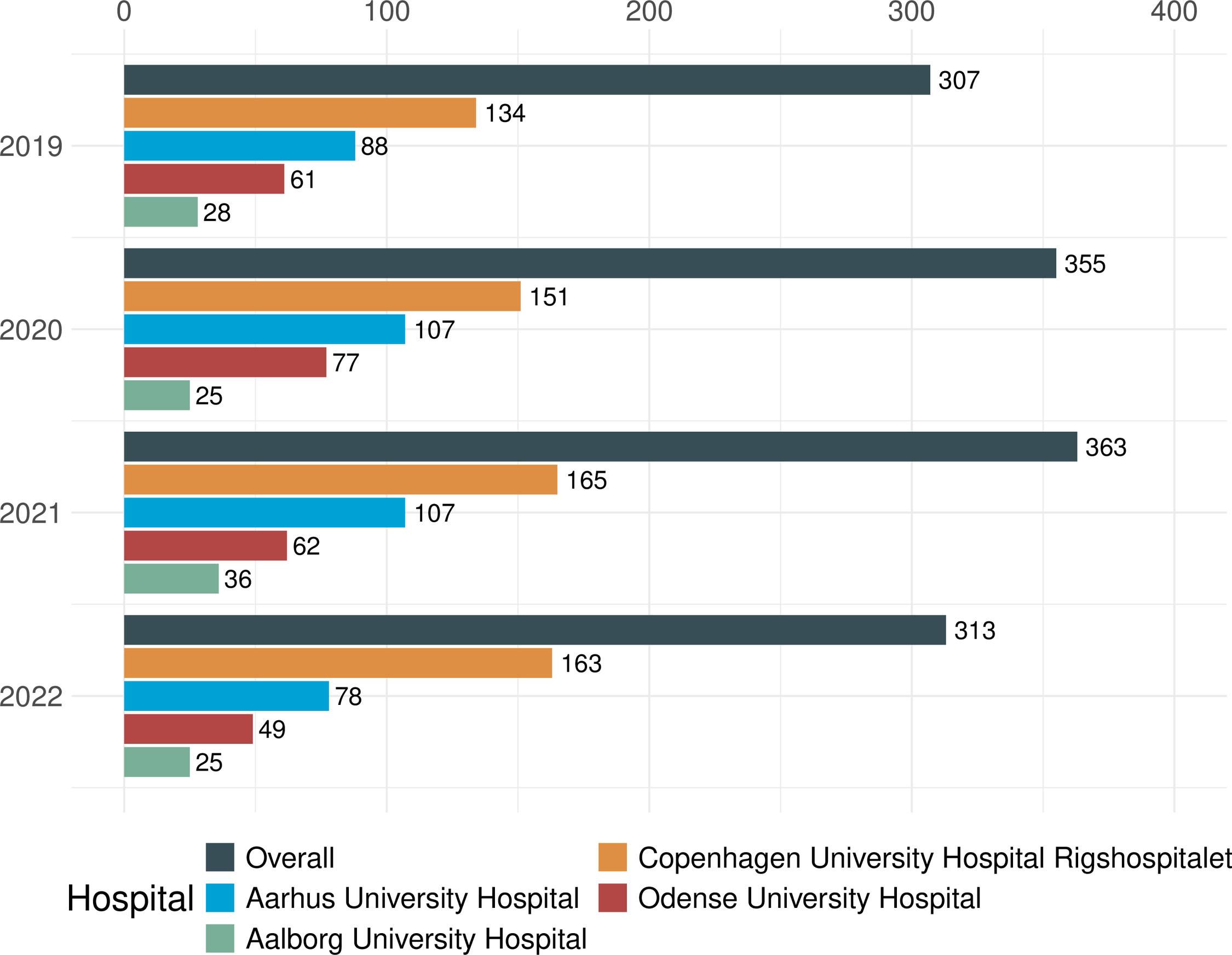

Supplement: S3 Fig — No decrease in the number of adolescents was observed during the COVID-19 pandemic from 2020 to 2021. Note that the individuals may be associated with multiple hospitals, so the totals do not sum across hospitals. (TIF) [file pone.0339721.s006.tif]
