# Supplementary material for: Defining the population of adolescents in need of comprehensive transitional care based on diagnosis, visit frequency, and disease complexity
Source: PLoS One. 2026 Jan 27;21(1):e0339721. doi: 10.1371/journal.pone.0339721 (PMC12843535; doi:10.1371/journal.pone.0339721)
Supplement: S3 Table — Each department is presented with a hospital code and department code for identification in the Danish National Patient register. (DOCX) [file pone.0339721.s004.docx]

**S3 Table. List of hospitals in Denmark with a pediatric department.**

| **Hospital** | **Hospital code** | **Department code** |
| --- | --- | --- |
| Copenhagen University Hospital Rigshospitalet, Department of Pediatrics and Adolescent Medicine | 1301 | 32 |
| Copenhagen University Hospital Rigshospitalet, Department of Neonatology | 1301 | 47 |
| Copenhagen University Hospital Rigshospitalet, Department of Growth and Reproduction | 1301 | 61 |
| Copenhagen University Hospital Rigshospitalet, Department of Clinical Genetics | 1301 | 80 |
| Copenhagen University Hospital Rigshospitalet, Department of Pediatric Surgery | 1301 | 87 |
| Hvidovre Hospital | 1330 | 60 |
| Herlev Hospital | 1516 | 37 |
| Nordsjællands Hospital, Hillerød | 2000 | 10 |
| Holbæk Hospital | 3800 | H8 |
| Nykøbing Falster Hospital | 3800 | V9 |
| Zealand University Hospital, Roskilde | 3800 | B0 |
| Slagelse Hospital | 3800 | N9 |
| Odense University Hospital | 4202 | 25 |
| Aabenraa Hospital | 5000 | 23 |
| Esbjerg and Grindsted Hospital | 5501 | 02H, 046, 04B, 22H |
| Kolding Hospital | 6007 | 11 |
| Aarhus University Hospital | 6620 | 24 |
| Viborg Regional Hospital | 6630 | 09 |
| Gødstrup Regional Hospital (Herning) | 6640 | 24 |
| Randers Regional Hospital | 7005 | 15 |
| Aalborg University Hospital | 8001 | 25 |
| Hjørring Regional Hospital | 8003 | 16 |

Each department is presented with a hospital code and department code for identification in the Danish National Patient register.
