# Supplementary material for: Defining the population of adolescents in need of comprehensive transitional care based on diagnosis, visit frequency, and disease complexity
Source: PLoS One. 2026 Jan 27;21(1):e0339721. doi: 10.1371/journal.pone.0339721 (PMC12843535; doi:10.1371/journal.pone.0339721)
Supplement: S4 Table — Most common primary diagnosis (3-digit ICD-10 codes) per visit for individuals included in specificity step 1 (Individuals aged 12–17 years with at least one outpatient visit at a tertiary Danish hospital during 2019–2022) and excluded in step 2 (Individuals aged 12–17 years with at least one outpatient visit at a tertiary Danish hospital with a diagnosis associated with an expected need for transitional care during 2019–2022), as the diagnose related to the tertiary hospital visit is not qualified as a need of transition diagnosis. N is the number of visits with the diagnosis listed, and the total number of outpatient visits is 177,583. (DOCX) [file pone.0339721.s007.docx]

**S4 Table** **Diagnoses associated with exclusion from the final study population**

|  | **ICD-10** |  | **N** |
| --- | --- | --- | --- |
| 1 | Z01 | Encounter for general examination without complaint, suspected or reported diagnosis | 53,231 (30.0%) |
| 2 | Z09 | Encounter for follow-up examination after completed treatment for conditions other than malignant neoplasm | 8,517 (4.8%) |
| 3 | Z03 | Encounter for medical observation for suspected diseases and conditions ruled out | 7,091 (4.0%) |
| 4 | M41 | Scoliosis | 4,227 (2.4%) |
| 5 | Z50 | Contacts regarding rehabilitation | 3,684 (2.1%) |
| 6 | Q37 | Cleft palate with cleft lip | 3,415 (1.9%) |
| 7 | S83 | Dislocation and sprain of joints and ligaments of knee | 3,198 (1.8%) |
| 8 | K07 | Dento-facial anomalies | 3,102 (1.7%) |
| 9 | S62 | Fracture at wrist and hand level | 3,078 (1.7%) |
| 10 | S52 | Fracture of forearm | 2,745 (1.5%) |

Most common primary diagnosis (3-digit ICD-10 codes) per visit for individuals included in specificity step 1 (Individuals aged 12 to 17 years with at least one outpatient visit at a tertiary Danish hospital during 2019-2022) and excluded in step 2 (Individuals aged 12 to 17 years with at least one outpatient visit at a tertiary Danish hospital with a diagnosis associated with an expected need for transitional care during 2019-2022), as the diagnose related to the tertiary hospital visit is not qualified as a need of transition diagnosis. N is the number of visits with the diagnosis listed, and the total number of outpatient visits is 177,583.
