# Supplementary material for: Defining the population of adolescents in need of comprehensive transitional care based on diagnosis, visit frequency, and disease complexity
Source: PLoS One. 2026 Jan 27;21(1):e0339721. doi: 10.1371/journal.pone.0339721 (PMC12843535; doi:10.1371/journal.pone.0339721)
Supplement: S5 Table — Complexity is categorized as severity A to D, with A being minimum expected need for transition, and D maximum. If an individual has more than one diagnosis requiring transition of care, they are included in the most severe group they qualify for. Two scenarios are represented, “lower bound” scenarios refer to the minimal expected need of transition care applied to a diagnosis, “upper bound” refers to the maximum. Individuals are represented as unique per hospital, and 14–15 years of age by the time of the hospital contact. (DOCX) [file pone.0339721.s008.docx]

**S5 Table. Distribution of unique adolescents per hospital, 14- to 15-year-old, in the categorization of degree of complexity for surgical diagnosis.**

| **Disease complexity**  **(A-D)** | **Overall**  **(All Danish tertiary hospitals)** | | **Copenhagen University**  **Hospital Rigshospitalet** | | **Aarhus**  **University**  **Hospital** | | **Odense**  **University**  **Hospital** | | **Aalborg**  **University**  **Hospital** | |
| --- | --- | --- | --- | --- | --- | --- | --- | --- | --- | --- |
|  | *Lower bound* | *Upper bound* | *Lower bound* | *Upper bound* | *Lower bound* | *Upper bound* | *Lower bound* | *Upper bound* | *Lower bound* | *Upper bound* |
| **A** | 328 | 186 | 158 | 82 | 69 | 37 | 61 | 29 | 45 | 40 |
| **B** | 1,139 | 1,137 | 412 | 417 | 405 | 413 | 181 | 163 | 160 | 159 |
| **C** | 514 | 202 | 286 | 136 | 143 | 36 | 109 | 55 | 13 | < 5 |
| **D** | - | 456 | - | 221 | - | 131 | - | 104 | - | 17 |

Complexity is categorized as severity A to D, with A being minimum expected need for transition, and D maximum. If an individual has more than one diagnosis requiring transition of care, they are included in the most severe group they qualify for. Two scenarios are represented, “lower bound” scenarios refer to the minimal expected need of transition care applied to a patients diagnose, “upper bound” refer to the maximum. Individuals are represented as unique per hospital, and 14 to 15 years of age by the time of the hospital contact.
