# Supplementary material for: Defining the population of adolescents in need of comprehensive transitional care based on diagnosis, visit frequency, and disease complexity
Source: PLoS One. 2026 Jan 27;21(1):e0339721. doi: 10.1371/journal.pone.0339721 (PMC12843535; doi:10.1371/journal.pone.0339721)
Supplement: S6 Table — Representation of the ten most common transition diagnoses (3-digit ICD-10 codes) for individuals with at least two visits at tertiary hospitals with diagnoses associated with an expected need for transitional care while aged 12–17 years, with at least one visit at age 16- to17-years -old. Numbers (N) are unique individuals with an outpatient visit with the diagnosis, percentages relative to the overall number of unique individuals. (DOCX) [file pone.0339721.s009.docx]

**S6 Table. The top ten transition diagnoses for the tertiary population for all individuals across tertiary hospitals**

|  | **ICD-10** |  | **N (%)** |
| --- | --- | --- | --- |
| **Overall** (N = 4,677) | | | |
| 1 | M08 | Juvenile arthritis | 574 (12.3%) |
| 2 | J45 | Asthma | 391 (8.4%) |
| 3 | E10 | Type 1 diabetes mellitus | 387 (8.3%) |
| 4 | G40 | Epilepsy | 270 (5.8%) |
| 5 | G80 | Cerebral palsy | 166 (3.5%) |
| 6 | K90 | Intestinal malabsorption | 146 (3.1%) |
| 7 | Q87 | Other specified congenital malformation syndromes affecting multiple systems | 139 (3.0%) |
| 8 | K50 | Crohn disease (reginal enteritis) | 137 (2.9%) |
| 9 | Q21 | Congenital malformations of cardiac septa | 111 (2.4%) |
| 10 | Z94 | Transplanted organ and tissue status | 109 (2.3%) |
| **For Copenhagen University Hospital Rigshospitalet** (N = 1,641) ^a^ | | | |
| 1 | M08 | Juvenile arthritis | 276 (16.8%) |
| 2 | G40 | Epilepsy | 70 (4.3%) |
| 3 | Z94 | Transplanted organ and tissue status | 69 (4.2%) |
| 4 | J45 | Asthma | 67 (4.1%) |
| 5 | Q87 | Other specified congenital malformation syndromes affecting multiple systems | 67 (4.1%) |
| 6 | Q21 | Congenital malformations of cardiac septa | 57 (3.5%) |
| 7 | Q20 | Congenital malformations of cardiac chambers and connections | 48 (2.9%) |
| 8 | Q25 | Congenital malformations of great arteries | 45 (2.7%) |
| 9 | Q85 | Phacomatoses, not elsewhere classified | 44 (2.7%) |
| 10 | E78 | Disorders of lipoprotein metabolism and other lipidemia | 42 (2.6%) |
| **For Odense University Hospital** (N = 1,298) | | | |
| 1 | J45 | Asthma | 215 (16.6%) |
| 2 | E10 | Type 1 diabetes mellitus | 138 (10.6%) |
| 3 | M08 | Juvenile arthritis | 99 (7.6%) |
| 4 | G40 | Epilepsy | 84 (6.5%) |
| 5 | K90 | Intestinal malabsorption | 64 (4.9%) |
| 6 | K50 | Crohn disease (reginal enteritis) | 54 (4.2%) |
| 7 | G80 | Cerebral palsy | 47 (3.6%) |
| 8 | K51 | Ulcerative colitis | 34 (2.6%) |
| 9 | Q85 | Phacomatoses, not elsewhere classified | 26 (2.0%) |
| 10 | Q87 | Other specified congenital malformation syndromes affecting multiple systems | 26 (2.0%) |
| **For Aarhus University Hospital** (N = 1,204) | | | |
| 1 | M08 | Juvenile arthritis | 246 (20.4%) |
| 2 | E10 | Type 1 diabetes mellitus | 129 (10.7%) |
| 3 | G40 | Epilepsy | 63 (5.2%) |
| 4 | G80 | Cerebral palsy | 53 (4.4%) |
| 5 | K50 | Crohn disease (reginal enteritis) | 49 (4.1%) |
| 6 | Q87 | Other specified congenital malformation syndromes affecting multiple systems | 42 (3.5%) |
| 7 | K90 | Intestinal malabsorption | 39 (3.2%) |
| 8 | K51 | Ulcerative colitis | 33 (2.7%) |
| 9 | J45 | Asthma | 32 (2.7%) |
| 10 | Q21 | Congenital malformations of cardiac septa | 30 (2.5%) |
| **For Aalborg University Hospital** (N = 588) | | | |
| 1 | E10 | Type 1 diabetes mellitus | 114 (19.4%) |
| 2 | J45 | Asthma | 76 (12.9%) |
| 3 | G40 | Epilepsy | 55 (9.4%) |
| 4 | G80 | Cerebral palsy | 34 (5.8%) |
| 5 | K90 | Intestinal malabsorption | 33 (5.6%) |
| 6 | M08 | Juvenile arthritis | 30 (5.1%) |
| 7 | K50 | Crohn disease (reginal enteritis) | 24 (4.1%) |
| 8 | E23 | Hypofunction and other disorders of pituitary gland | 23 (3.9%) |
| 9 | K51 | Ulcerative colitis | 18 (3.1%) |
| 10 | M65 | Synovitis and tenosynovitis | 17 (2.9%) |

Representation of the ten most common transition diagnoses (3-digit ICD-10 codes) for individuals with at least two visits at tertiary hospitals with diagnoses associated with an expected need for transitional care while aged 12 to 17 years, with at least one visit at age 16- to17-years -old. Numbers (N) are unique individuals with an outpatient visit with the diagnosis, percentages relative to the overall number of unique individuals.

^a^*Individuals with diabetes (T1D) and inflammatory bowel disease (IBD) are not followed up at Copenhagen University Hospital Rigshospitalet. Additionally, the catchment area is very small compared to, for example, Aarhus*
